# Lateral Violence in Nursing Survey: Instrument Development and Validation

**DOI:** 10.3390/healthcare5030033

**Published:** 2017-07-19

**Authors:** Lynne S. Nemeth, Karen M. Stanley, Mary M. Martin, Martina Mueller, Diana Layne, Kenneth A. Wallston

**Affiliations:** 1 College of Nursing, Medical University of South Carolina, Charleston, SC 29425, USA; karenstanley1978@gmail.com (K.M.S.); drmarymartin@aol.com (M.M.M.); muellerm@musc.edu (M.M.); layne@musc.edu (D.L.); 2 School of Nursing, Vanderbilt University, Nashville, TN 37235, USA; ken.wallston@vanderbilt.edu

**Keywords:** horizontal violence, bullying, oppressed group behavior, construct validity

## Abstract

An examination of the psychometric properties of the Lateral Violence in Nursing Survey (LVNS), an instrument previously developed to measure the perceived incidence and severity of lateral violence (LV) in the nursing workplace, was carried out. Conceptual clustering and principal components analysis were used with survey responses from 663 registered nurses and ancillary nursing staff in a southeastern tertiary care medical center. Where appropriate, Cronbach’s alpha (α) evaluated internal consistency. The prevalence/severity of lateral violence items constitute two distinct subscales (LV by self and others) with Cronbach’s alpha of 0.74 and 0.86, respectively. The items asking about potential causes of LV are unidimensional and internally consistent (alpha = 0.77) but there is no conceptually coherent theme underlying the various causes. Respondents rating a potential LV cause as “major” scored higher on both prevalence/severity subscales than those rating it a “minor” cause or not a cause. Subsets of items on the LVNS are internally reliable, supporting construct validity. Revisions of the original LVNS instrument will improve its use in future work.

## 1. Introduction

The Institute of Medicine (IOM) reports on patient safety [1,2] and the Joint Commission standard related to disruptive and inappropriate behavior [3] increased the urgency to know the causes, prevalence and severity of negative behaviors in healthcare. The American Nurses Association published a position statement in 2015 on incivility, bullying and workplace violence citing the professional responsibility of nurses to display ethical and civil behavior as addressed in the Nurses’ Code of Ethics as a primary driver for the development of this position [4]. Healthcare environments also present unique challenges due to the potential risk of violence from patients to caregivers [5]. Disruptive and negative behaviors displayed by nurses, physicians and other healthcare workers have been documented by researchers such as Quine [6], Farrell and Shafiei [7], Hutchinson et al. [8], Manderino and Berkey [9], and O'Daniel and Rosenstein [10], and Walrath et al. [11,12].

The American Nurses Association [4,13,14] established a strong position on lateral violence in nursing, subsequent to increased reporting in the nursing literature over the past two decades. “Lateral violence refers to acts that occur between colleagues, where bullying is described as acts perpetrated by one in a higher level of authority and occur over time” [15]. Descriptive studies have addressed negative nurse behaviors and their impact on unit tone, retention, and cost to nursing [16,17,18,19,20,21,22,23,24,25,26,27,28]. Psychological and emotional consequences for the recipients of lateral violence (LV) are well documented, including decreased self-esteem, passion for the profession [29], depression, self-hatred, and feelings of powerlessness [29,30]. Qualitative analysis of nurses’ experience with lateral violence showed that nurses who experience LV try to “make things right” by understanding the situation surrounding the event, assessing the situation, taking action, and finally judging the outcomes [31]. Estimates of prevalence of disruptive behavior reported within the literature vary depending on displayed behavior and setting. Verbal abuse in the presence of patients’ occurs as little as 7% of the time within the perioperative setting, to as often as 34% of the time within the emergency department, 34% in nursing students, and 43% in clinical settings [32,33,34]. Magnavita and Heponiemi [35] identified that one in ten healthcare workers experience some form of physical or non-physical violence over twelve months. Many nurses are engaged in ending lateral violence as the norm, crave a healthy work environment, and recognize the power differential within the work environment that potentially leads to feelings of oppression [36]. Consequences of LV for novice nurses include decreased productivity, and consideration for leaving the profession [37,38]. While these studies provide evidence of the seriousness of the problem and the susceptibility of the profession to LV, interventions have rarely been studied in the settings where nurses work [39,40,41].

### 1.1. Background and Conceptual Framework

Freire [42] used oppression theory in his study of the effect of cultural dominance on behavior in the context of colonized South Americans, and his work was crucial to Roberts [43] application of oppression theory to nursing behavior. Several other nurse scholars have since used oppression theory to explain, at least in part, the occurrence of LV in the nursing profession [19,20,25,39,40,44]. Oppression theory, powerlessness and the health belief model framed the development of the Lateral Violence in Nursing Survey (LVNS). Two of the authors (KS and MMM) both with expertise in psychiatric mental health nursing, and nursing administration developed the items, which were reviewed by another author of this paper (LSN) (a nursing research mentor), and a nursing administrator for clarity and construct validity. Several items for the LVNS were drawn from oppression theory [39,42,43] related to nursing. The health belief model [45] provided the concepts of susceptibility (to either being a victim or a perpetrator) and seriousness (understanding the impact of one’s behavior on the recipient). These two concepts provided cognitive, psychological and behavioral content for developing individual items.

### 1.2. Instrument Develeopment

Concepts from the nursing research that influenced development of the Lateral Violence in Nursing Survey (LVNS) are presented in Table 1. These studies used instruments or techniques that provided information about the types of negative behaviors that nurses direct toward one another and the effect that this aggression has on individuals and systems. Griffin [39] examined two decades of nursing literature and described ten frequently reported forms of LV. These descriptions were particularly important to our study because they enabled participants to put a name to the negative experiences they have suffered at the hands of nurse colleagues. No instruments that measured the prevalence, severity and causes of LV in a variety of clinical settings by nurse researchers with varied skill levels, that is, novice to expert, were available at the time this instrument was developed.

### 1.3. Instrument Administration

To this end, the Lateral Violence in Nursing Survey (LVNS) was developed and administered in July 2005 as a web-based survey to over 1850 nursing personnel in a large academic medical center [40]. The LVNS consists of a 23-item survey focusing on the prevalence and seriousness of LV, causes and other aspects of LV within the workplace. Table 2 provides specific questions from the survey focused on the prevalence and seriousness of LV, Table 3 displays specific questions related to causes of LV and Table 4 details specific questions related to other aspects of LV within the workplace. The initial data analysis included both quantitative (SPSS^®^, IBM Corporation, Somers, NY, USA) and qualitative (NVivo, QSR International Pty, Doncaster, Victoria, Australia) findings describing the nature of the problem and potential mediating factors [40]. The purpose of this paper is to report psychometric qualities of LVNS items establishing reliability and validity of this tool for use within large academic medical centers.

## 2. Materials and Methods

The aim of this study was to identify the factor structure and report the psychometric characteristics of the LVNS. An exploratory factor analysis estimated the variability due to common factors among the observed variables included within this survey. Along with demographic/background questions, the survey contained a number of questions related to frequency, seriousness and potential causes of lateral violence in nursing plus one question regarding respondents’ observations of interdisciplinary violent behavior.

### 2.1. Data Collection, Instrument Scoring and Sample

This hospital intranet-based survey requiring no personal identifiers by respondents was available for a three-week duration. An email invitation was sent to 1850 nursing personnel (included all levels of nursing personnel and management), which yielded 663 usable responses. Literature-based definitions of LV behaviors, potential causes of LV, and perceptions of the severity of LV were used to formulate the items. The response options for the behaviors and perceived severity items included ordinal scales such as “often” to “never” or “very serious” to “not serious at all” and respondents had the option not to respond to these items. For the items about possible causes of the LV, the respondents were asked to indicate if this was “a major cause, “a minor cause,” “not a cause,” or they could indicate if they were “not sure” or “did not wish to respond.” Four open-ended items provided participants with the opportunity to clarify their quantitative responses in their own words. All respondents were anonymous. The Institutional Review Board of the Medical University of South Carolina approved this study.

### 2.2. Data Analysis

Preliminary data verifications and manipulations focused on developing consistent coding of missing data including don’t know, refused, not sure, and not applicable responses. A consistent variable coding strategy was identified for item responses where lower values represent less lateral violence (or its concomitants) and higher values represent more lateral violence. Resulting coding schemes were verified using descriptive statistics; pairwise cross-tabulations of coded item responses; and Pearson, Spearman, and polychoric correlation analyses.

Initial logical analyses of items resulted in a classification of items into several different “item clusters” that had consensual validity among the original investigators. One cluster included items that asked about the prevalence/seriousness of lateral violence in nursing (see Table 2); a second cluster included items that asked about potential causes of lateral violence in nursing (see Table 3) and a third cluster contained items that asked about other aspects of lateral violence in the workplace (see Table 4). The remainder of the survey consisted of items related to socio-demographic and professional characteristics of the participants (i.e., sex, age, race/ethnicity, current job category, years of experience in job category, years of experience in job category at this institution, and area of nursing practice).

Exploratory principal components factor analysis was used to investigate the underlying structure among survey variables; one-way analysis of variance (ANOVA) was used to examine relationships between survey variables. Due to the multiple ANOVAs conducted, a Bonferroni correction was applied to adjust the Type I error rate to *p* < 0.003 for the F-statistic (see Table 5). Analyses were carried out in SPSS version 21 (SPSS^®^, IBM Corporation, Somers, NY, USA).

## 3. Results

### 3.1. Sample

Registered nurses (601) comprised 91% of the total 663 study participants. Participants ranged in age from 20 to 70 years and were predominantly female (91%) and white (82%). The majority (60%) of the participants had from six to 30 years of experience in their job category and worked in inpatient and outpatient settings throughout the medical center.

### 3.2. Lateral Violence Causes Scale

The most highly endorsed “major” potential causal explanations for LV had to do with stress related to inadequate staffing or resources to handle the workload (Q15), a general societal decline in civil behavior (Q19), and major personality clashes among a few people (Q18). The causal explanations for LV that received the least endorsement were cultural misunderstanding (Q12) and professional behavior not being stressed in the workplace (Q17). On their face, the eight nursing lateral violence causes items (Qs 12–19) do not form a coherent, well-defined, internally consistent scale reflecting a common underlying phenomenon. Nevertheless, a principal components analysis of these eight items resulted in a single underlying factor with a Cronbach’s alpha of 0.77.

Further reflection on the content of these causal explanations suggests that there is no necessary reason why endorsement of one cause ought to be related to endorsement of other causes. Therefore, rather than trying to treat this as an internally consistent lateral violence causes scale, we decided that the various causes should be used individually to help understand perceived sources and geneses of the instances of nursing lateral violence that are captured by the prevalence/severity scales.

### 3.3. Relationship among Possible Causes and Prevalence/Severity of LV by Self or Others

We conducted a series of one-way analyses of variance (ANOVAs) to explore the relationships between ratings of possible causes of the violence and scores on the prevalence/severity subscales. Each of the potential causes (Q12 through Q19) was explored in two separate analyses. In the first analysis, the dependent variable was the respondent’s score on the prevalence/severity of LV by self-subscale; in the second it was the subscale relating to LV by others. For the independent variable, respondents were classified according to whether they thought the explanation provided in the item was “not a cause of LV,” “a minor cause of LV,” or “a major cause of LV”. Table 5 presents the findings from these ANOVAs. As can be seen in the table, for 14 of the 16 analyses there were significant differences (*p* < 0.003) in prevalence/severity of LV scores depending on respondents’ ratings of whether the potential cause was responsible for the LV. The self and other’s LV ratings of prevalence/severity were highest for explanations seen to be the major causes and lowest for explanations that were not judged to be causes of LV.

### 3.4. Relationship among Possible Causes and Prevalence/Severity of LV by Self or Others

As is the case with the possible causes of LV items, the remaining items (Qs 6, 7, 10, 11, 20, 21 and 22) subsumed under the other aspects of lateral violence category in Table 4 would not be expected to form a coherent scale because there is no single latent factor that would underlie responses to each of these items. Therefore, no attempt was made to compute a Cronbach’s alpha for these items. Individually, however, they too may add detailed information about the experience of nursing lateral violence in particular settings, and the effectiveness of past training efforts.

## 4. Discussion

Psychometric studies of nursing workforce surveys are lacking in the literature. The Negative Acts Questionnaire (NAQ) [46] which was developed in Norway measures perceived exposure to bullying at work and has shown a Cronbach’s α of 0.92 in the English version. Bullying, however, is a distinct behavior defined by the author of that instrument as occurring at least twice weekly for six months or longer [47]. The NAQ measured the construct of bullying in studies by Simons [20] and Johnson and Rea [23]. The Nurse Workplace Behavior Scale (NWS) developed by DeMarco et al. [48] focuses on oppressed group behavior, and found a two-factor solution measuring oppressed self and oppressed group with Cronbach’s α at 0.81 and 0.78, respectively. This compares similarly to our self and other persons factor, but NWS questions focused specifically on characteristics of oppression, while our survey focused on the prevalence and perceptions of the seriousness of the behavior.

Vessey, Demarco, Gaffney and Budin [18] 30-item questionnaire examined the concept of bullying in nursing, provided data about which nurses were bullied, who were perpetrators, how long bullied nurses had been practicing, what effect bullying had on intent to leave, and what was done to address the bullying. No information was provided regarding the validity or reliability of the instrument used in this study. In their qualitative study of registered nurses’ experience with disruptive clinician behavior, Walrath [11] identified three types of disruptive behaviors: incivility, psychological aggression, and physical violence. Findings from Walrath’s study led to the development of a new survey instrument, the Disruptive Clinician Behavior Survey for Hospitals, which was used to conduct an organizational assessment of disruptive behaviors. The Disruptive Clinician Behavior Survey assessed the scope, responses to, and impact of disruptive behavior across three clinician groups (all levels of clinical and administrative nurses, certified nurse midwives, certified nurse anesthetists, physician assistants, full-time clinical faculty, fellows, and house staff) [12]. The Disruptive Clinical Behavior Survey for Hospitals developed by Walrath and colleagues [12] is a 62-item survey with a 1-factor solution for each of the six subscales, overall survey reliability reported as Cronbach’s α of 0.93, with subscales ranging in value from 0.72 to 0.92 [10].

Roberts, et al. [49] concluded that the LVNS instrument is the only published tool that can be used to measure prevalence and severity in the field of nursing. All above instruments share an intention to measure bullying to demonstrate the degree of impact for intervention development to address the negative impacts of these behaviors in the nursing workforce.

Nursing research on the topic of lateral violence began before the Joint Commission (JC) leadership standard 3.01.01 [3] and the IOM report “To Err is Human” [1]. This reinforces the important implications workforce disruptions have as they relate to patient safety. As a result, the need for data-driven information about inappropriate and disruptive behavior by the healthcare workforce is even more critical. Taken individually, the potential causes of lateral violence identified on the LVNS (Q12 through Q19) may provide important clues to the mitigation of lateral violence apparent in specific settings. Identifying and addressing the causes will result in a decrease in healthcare system tolerance for unprofessional and disruptive behavior so that the interprofessional team may more easily meet patient safety goals.

### Limitations

The LVNS was administered to a group of nursing staff and managers at all levels of the organization, which may have confounded the analyses. Nurse managers are often perpetrators of the behaviors observed in this survey. This study did not evaluate confirmatory factory analysis. The response rate of this electronic survey was 36%, limiting the generalizability of the findings. This report does not intend to infer the true prevalence of lateral violence in the population of nurses. The survey results represent one southeastern United States academic medical center, and findings may differ in other regions or in smaller community hospitals. Yet, due to the lack of valid surveys to measure the impact of lateral violence, we believe that the psychometric characteristics of this survey offer promise for future research and nursing workforce interventions.

## 5. Conclusions

The LVNS may provide nurse leaders with an evidenced-based tool to assist with retention, and developing a positive unit tone. Recognizing that nurse managers are the key personnel to establish and maintain a positive unit tone may enable hospital administrators to support nurse managers through development of a unit- and hospital-wide culture that fosters zero tolerance for lateral violence. The LVNS can validate the presence and seriousness of lateral violence on a nursing unit or within an entire nursing service. Armed with this evidence, any expense associated with using interventions to mitigate the effects of LV on retention, patient safety, and overall staff satisfaction can more effectively be justified.

## Figures and Tables

**Table 1:** Nursing research that influenced development of the Lateral Violence in Nursing (LVNS) instrument.

First Author, (Year), Sample Size & Location	Dimensions and/or Outcome Measures	Research Design	Reliability/Validity	Strengths	Limitations	Comments
DeMarco (2002) *n* = 115 USA	Measurement of behaviors between work and home; measurement of experience of self-advocacy within the work group	Descriptive correlational design using the Staff Nurse Workplace Behaviors Scale (SNWBS) and Silencing the Self Scale-Work (STSS-Work)	SNWBS was shown to be a valid and reliable instrument; STSS-Work alpha scores of internal consistency range from 0–86 to 0–94 and test–retest reliability from 0–88 to 0–93	Solid research design with integration of theoretical concepts using a systematic design	Complexity of study requires extensive research skills and experience to replicate	Statistically significant relationships found between workplace, family and silencing behaviors
Dunn (2003) *n* = 145 USA	Examined relationship between the presence of sabotage in an OR and nurses’ job satisfaction levels	Descriptive correlational design using Briles’ Sabotage Savvy Questionnaire and the Index of Work Satisfaction Questionnaire	Validity and reliability established for both instruments	Application of cognitive dissonance theory to the understanding of why nurses tolerate aggressive colleague behaviors	29% response rate; very long questionnaires; cannot generalize to other populations	Sabotage found to be common in the OR setting, but did not correlate with low job satisfaction. Cognitive dissonance theory applied
Farrell (1999) *n* = 270 Australia	Examined type, level and natures of aggression; nurses’ action post aggression; most distressing type of aggression	Questionnaire Survey	No details provided	Began to quantify the extent of aggression toward nurses and its effect on them	Random sampling not employed; cannot generalize to other populations	Aggression from nurse colleagues was identified as most distressing to nurses
Griffin (2004) *n* = 26 USA	Examined effect of education about lateral violence and cognitive rehearsal techniques on new graduate nurses	Exploratory descriptive study with an applied intervention; three focus groups were videotaped responding to open-ended questions one year after the intervention	No details provided	Definitions of ten common forms of lateral violence were provided; intervention for nurses use in the work setting applied	Small *n*	“Overall, the retention rate in this study population was positively affected” (p. 257)
McKenna (2003) *n* = 551 New Zealand	Assessed the prevalence, characteristics, psychological impact of horizontal violence on first year nurses and adequacy of training to manage horizontal violence	Descriptive study using the Impact of Event Scale, a questionnaire modified from one developed by Coverdale et al., 2001 to explore the nature and impact of interpersonal conflict among nursing colleagues	No details provided for first questionnaire; Impact of Event Scale is a “…validated and reliable measure of subjective psychological distress” (p. 92)	Increased awareness of extent of the aggression directed toward new nurses and its impact on individuals and the nursing profession	Study sample not representative on variable of ethnicity	Nearly half of the events described as distressing to nurses in their first year of practice were not reported; more than one-third of the nurses said they had training in their undergraduate program in coping with interpersonal conflict
Quine (1999) *n* = 1100 England	Inventory of Bullying Behaviors; Job Induced Stress Scale; Hospital Anxiety and Depression Scale; Propensity to Leave Scale	Questionnaire Survey	No details provided	Large *n* 70% response rate	Complexity of data from multiple scales; cannot generalize to US settings	Support at work was found to be a protective factor from the negative effects of bullying
Skillings (1992) *n* = 6 USA	Three outcome themes: nurses experience multidimensional and socially constructed oppression; horizontal violence is an expression of oppressed group behavior in nursing; overcoming oppression involves a process of consciousness raising and transformation	Taped, semi-structured individual meetings; one group meeting	No details provided	Process of study itself promoted nurse unity and support	Small *n* Difficult to replicate; not generalizable to other settings	Nurses were given a voice by encouraging them to address the negative aspects of nurse relationships

**Table 2:** Items that asked about the prevalence/seriousness of lateral violence in nursing.

Q#	Question Text	Response Options
Q1:	How often are you treated with courtesy and respect by coworkers?	*Often, Sometimes, Rarely, Never*
Q2:	Compared with other causes of stress and tension related to your job, would you put lateral violence:	*among Top, Middle, Bottom*
Q3:	Would you say that lateral violence toward coworkers in your work area is:	*Widespread or caused by many, Limited to a few, Not a problem*
Q4:	How serious a problem would you say lateral violence toward coworkers is in your work area?	*Very serious, Somewhat serious, Not too serious, Not serious at all*
Q5:	How often do you see coworkers losing their patience and directing behaviors that can be interpreted as lateral violence toward coworkers?	*Often, Sometimes, Rarely, Never*
Q8:	How often do you find yourself losing your patience and being less polite to coworkers than you would want to be?	*Often, Sometimes, Rarely, Never*
Q9:	How often have you crossed the line and used behaviors that would be interpreted as lateral violence toward a coworker?	*Often, Sometimes, Rarely, Never*
Q23:	This survey is designed to assess lateral violence within nursing. However, the negative behaviors identified in the survey introduction may also occur among members of the interdisciplinary team. Would you say this problem is:	*Very serious, Somewhat serious, Not too serious, Not serious at all*

**Table 3:** Items that asked about the causes of lateral violence in nursing.

Q#	Question Text	Response Options
Q12:	Misunderstandings caused by cultural differences	*Major cause, Minor cause, Not a cause*
Q13:	Leaders and coworkers are not willing to intervene	*Major cause, Minor cause, Not a cause*
Q14:	Rude behavior is so common that coworkers adopt it	*Major cause, Minor cause, Not a cause*
Q15:	Stress related to inadequate staff and resources to handle the workload	*Major cause, Minor cause, Not a cause*
Q16:	New nurses being tested to see if they can make it in this work area	*Major cause, Minor cause, Not a cause*
Q17:	Professional behavior is not stressed in this work area	*Major cause, Minor cause, Not a cause*
Q18:	Major personality clashes among a few people	*Major cause, Minor cause, Not a cause*
Q19:	A decline in polite and respectful behavior in our society in general	*Major cause, Minor cause, Not a cause*

**Table 4:** Items that asked about other aspects of lateral violence in the workplace.

Q#	Question Text	Response Options
Q6:	When this happens, is it typically because the recipient was (check all that apply):	*[**Y**,**N**] Recipient was in a position perceived as powerless*
		*[**Y**,**N**] Recipient was unwilling to stand up to the coworker*
		*[**Y**,**N**] Recipient was not supported by others in the workplace*
		*[**Y**,**N**] Recipient was… something else…*
	*Q6. “Something else” please describe.*	
Q7:	How safe from retaliation would you feel reporting an episode of lateral violence?	*Very safe, Uneasy but still willing to report, Not safe*
Q10:	Have you received special training on techniques for dealing with rude or disrespectful persons?	*Yes, No*
Q10a:	If YES, how effective would you say this training has been?	*Very effective, Somewhat effective, Not too effective,* *Not effective at all*
Q11:	Have you personally observed a situation at work where lateral violence toward a coworker:	*Led to a physical confrontation, Threatened to escalate into physical confrontation,* *Have not personally observed either*
Q20:	If you have left a nursing position where lateral violence was a factor, what percentage of your decision to leave was related to your experience with lateral violence?	*100–75%,* *74–50%,* *49–25%,* *<25%* *Not applicable*
Q21:	Is there a recent incident involving lateral violence in your work area that you would like to share?	*Yes, No*
Q22:	Do you think something can be done in your work area to help solve problems related to lateral violence?	*Yes, No*
	*Q22a. If YES, This can be done, please describe:*	

**Table 5:** Mean scores on the Prevalence/Severity of LV Subscales as a Function of Endorsement of Causal Explanation of LV.

Causal Explanation for LV	Subscale	Not a Cause	A Minor Cause	A Major Cause	F-Value
Q12—Cultural misunderstandings	LV by Self	1.02 a,b	1.07 b,c	1.23 c	3.18 *
	LV by Others	1.38 a,b	1.51 b,c	1.85 c	12.53 ***
Q13—Unwillingness of leaders/coworkers to intervene	LV by Self	0.82 a	12.06b	1.20c	18.93 ***
	LV by Others	0.98 a	1.43 b	1.85 c	99.24 ***
Q14—Rude behavior is so common	LV by Self	0.90 a	0.99 b	1.32 b	26.59 ***
	LV by Others	0.98 a	1.46 b	2.00 c	160.06 ***
Q15—Stress due to inadequate staffing/resources	LV by Self	0.82 a	1.02 b	1.19 c	16.44 ***
	LV by Others	1.24 a	1.40 b	1.60 c	13.25 ***
Q16—“Testing” of new nurses to see if they can survive	LV by Self	0.95 a	1.08 a,b	1.19 b	7.20 **
	LV by Others	1.23 a	1.48 b	1.84 c	39.89 ***
Q17—Professional behavior not stressed in workplace	LV by Self	0.91 a	1.15 b	1.23 b	17.16 ***
	LV by Others	1.14 a	1.63 b	1.97 c	100.48 ***
Q18—Major personality clashes among a few people	LV by Self	0.66 a	1.07 b	1.17 b	22.46 ***
	LV by Others	0.77 a	1.41 b	1.76 c	81.82 ***
Q19—General societal decline in civil behavior	LV by Self	0.89 a	1.01 a	1.22 b	14.81 ***
	LV by Others	1.20 a	1.42 b	1.66 c	22.34 ***

Legend: a,b,c Means with a subscript in common are *not* significantly different from one another at *p* < 0.05 by the Tukey b post-test. * *p* < 0.05; ** *p* < 0.01; *** *p* < 0.003. LV: lateral violence.
